# Fisetin's Promising Antitumor Effects: Uncovering Mechanisms and Targeting for Future Therapies

**DOI:** 10.1055/s-0043-1772219

**Published:** 2023-08-09

**Authors:** Eskandar Qaed, Bandar Al-Hamyari, Ahmed Al-Maamari, Abdullah Qaid, Haneen Alademy, Marwan Almoiliqy, Jean Claude Munyemana, Murad Al-Nusaif, Jameel Alafifi, Eman Alyafeai, Mohammed Safi, Zhaohong Geng, Zeyao Tang, Xiaodong Ma

**Affiliations:** 1 State Key Laboratory of Applied Organic Chemistry, Key Laboratory of Nonferrous Metal Chemistry and Resources Utilization of Gansu Province, College of Chemistry and Chemical Engineering, Lanzhou University, Lanzhou, People's Republic of China; 2 School of Pharmacy and State Key Laboratory of Applied Organic Chemistry, Lanzhou University, People's Republic of China; 3 The Key Laboratory of Neural and Vascular Biology, The Key Laboratory of New Drug Pharmacology and Toxicology, Department of Pharmacology, Ministry of Education, Hebei Medical University, Shijiazhuang, People's Republic of China; 4 N.I. Pirogov Russian National Research Medical University, Russia; 5 Taiz University Faculty of Medicine and Health Science, Yemen; 6 Department of Pharmacy, Faculty of Medicine and Health Sciences, University of Science and Technology, Aden, Yemen; 7 Department of Translational Molecular Pathology, The University of Texas MD Anderson Cancer Center, Houston, Texas, United States; 8 Department of Neurology and Liaoning Provincial Key Laboratory for Research on the Pathogenic Mechanisms of Neurological Diseases, the First Affiliated Hospital, Dalian Medical University, Dalian, People's Republic of China; 9 School of Pharmaceutical Science, Wenzhou Medical University, Wenzhou, People's Republic of China; 10 Department of Pharmacy, Dalian Medical University, Dalian, People's Republic of China; 11 Department of Cardiology, 2nd Affiliated Hospital of Dalian Medical University, Dalian, People's Republic of China

**Keywords:** apoptosis, autophagy, cell cycle arrest, fisetin

## Abstract

**Background** Cancer remains a critical global health challenge and a leading cause of mortality. Flavonoids found in fruits and vegetables have gained attention for their potential anti-cancer properties. Fisetin, abundantly present in strawberries, apples, onions, and other plant sources, has emerged as a promising candidate for cancer prevention. Epidemiological studies linking a diet rich in these foods to lower cancer risk have sparked extensive research on fisetin’s efficacy.

**Objective** This review aims to comprehensively explore the molecular mechanisms of fisetin's anticancer properties and investigate its potential synergistic effects with other anticancer drugs. Furthermore, the review examines the therapeutic and preventive effects of fisetin against various cancers.

**Methods** A systematic analysis of the available scientific literature was conducted, including research articles, clinical trials, and review papers related to fisetin’s anticancer properties. Reputable databases were searched, and selected studies were critically evaluated to extract essential information on fisetin’s mechanisms of action and its interactions with other anticancer drugs.

**Results** Preclinical trials have demonstrated that fisetin inhibits cancer cell growth through mechanisms such as cell cycle alteration, induction of apoptosis, and activation of the autophagy signaling pathway. Additionally, fisetin reduces reactive oxygen species levels, contributing to its overall anticancer potential. Investigation of its synergistic effects with other anticancer drugs suggests potential for combination therapies.

**Conclusion** Fisetin, a bioactive flavonoid abundant in fruits and vegetables, exhibits promising anticancer properties through multiple mechanisms of action. Preclinical trials provide a foundation for further exploration in human clinical trials. Understanding fisetin’s molecular mechanisms is vital for developing novel, safe, and effective cancer prevention and treatment strategies. The potential synergy with other anticancer drugs opens new avenues for combination therapies, enhancing cancer management approaches and global health outcomes.

## Introduction

Due to population increase and aging, particularly in less developed countries, where approximately 82% of the world's population resides, cancer is a primary cause of mortality in more and less economically developed nations. 1 A normal cell gradually changes into a cancer cell through a multistep process called oncogenesis. Changes occur at the cellular, genetic, and epigenetic levels during this process. Because of these alterations, the cell may hyperproliferative and multiply indefinitely; it may also evade apoptosis, continue angiogenesis, become invasive, and undergo metastasis. 2

Despite improvements in diagnostic and therapeutic techniques over time, cancer is still a difficult problem. 1 This is primarily due to increasing urbanization and cancer-related lifestyle decisions, such as unhealthful eating patterns. Prostate cancer (PCa) in men and breast cancer in women are the next two top causes of cancer-related fatalities for men and women. 3 Although monotargeted medicines are presently accessible, one target may cause toxicity and unwanted effects. Targeting ineffectual, resistant, and rising treatment costs are further drawbacks of targeted medicines. Since cancer is a multifactorial illness, preventative and therapeutic strategies using drugs that can target numerous biochemical and molecular pathways may be necessary. This seems to be the most feasible way to reduce the prevalence and impact of the disease. 4 The goal of cancer chemotherapy, a rapidly developing area of preventive oncology, is to suppress, delay completely, or even reverse the process of carcinogenesis by using synthetic, pharmacological, or natural substances. 5 6 Safety, efficacy, simplicity of availability, affordability, the potential to overcome resistance to other conventional medicines, and the potential to replace anticancer pharmaceuticals are just a few benefits of employing natural agents for chemoprevention. 7

More than 4,000 plant metabolites comprise the class of plant chemicals known as flavonoids, which have a range of biological effects. For example, a flavonoid called fisetin (3,3′, 4', 7-tetrahydroxyflavone) is mostly found in fruits and vegetables such as apples, strawberries, cucumbers, and onions. 8 9 10 In both in vitro and in vivo illness models, fisetin can potentially have pleiotropic effects. In addition, fisetin impacts the proliferation of tumor cells by regulating several upstream kinases, transcription factors, and their regulators. It does this by targeting several intracellular signal pathway components. Here, the form of research is reviewed and the anticancer properties of the dietary flavonoid fisetin are discussed, focusing on its function in cell development, proliferation, and death.

## Fisetin's Antitumor Properties

### Fisetin Involvement in the Apoptosis

Fisetin-induced apoptosis is a form of caspase-mediated regulated cell death. In human epidermoid carcinoma A431 cells, fisetin therapy led to a decrease in the expression of antiapoptotic protein and an increase in the expression of the proapoptotic protein. In addition, the fraction of cells with reduced mitochondrial membrane potential also increased, indicating that fisetin-induced apoptosis also destroys mitochondria.

**Fig. 1:**
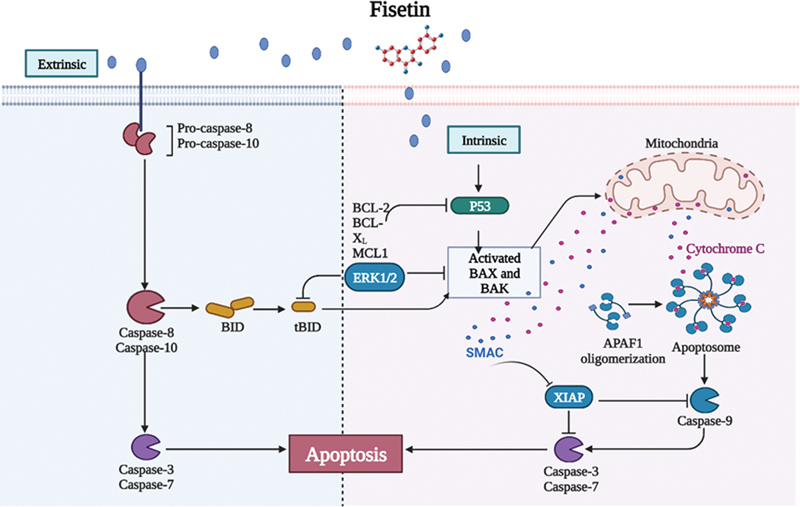
Induction of apoptosis. (1) Activation of caspase-8 by fisetin through extracellular, leading stimulating pro-caspase-8 to activate caspase-8, subsequently activating BID into tBID. Moreover, caspase-8 activates caspase-3, resulting in apoptosis. The occurrence of apoptosis can also be through intrinsic signal pathway, increasing the ratio of Bax/BAK and inhibiting Bcl-2 by fisetin through the activation of p53, which is regulated negatively by BCL-2, BCL-XL, MCL1; Meanwhile, ERK1,2 negatively regulates tBID and activates Bax/BAK, leading to the release of CytoC and over generation of ROS, which is also directly initiated by fisetin, the stimulation of AMPK and apoptosome formation under the effect of APAF, subsequently activating caspase-9 collectively, then activating caspase-3, leading to apopotosis, too. Besides both of caspase-3 and caspase-9 are inhibited by PARP, which is regulated negatively by SMAC/DIABLO. (2) Promotion of the release of Cyto C and the activation of caspase-3 and 9; (3) Upregulation of the production of ORS and activation of AMPK, leading to the production of Cyto C. Bid, AIF and the increase of the ratio of Bax to Bcl-2, causing the activation of caspase 3–9, leading to the upregulation of the production of apoptosis. “↓” indicates “to stimulate” “⊥” indicates “inhibit”; solid line represents the action directly; dotted line represents the action through several processes. Abbreviations: Bax, B cell lymphoma 2-associated X protein; MCL1, myeloid cell leukemia-1, BCL2 Family Member; Cyto C, cytochrome c; ROS, Reactive oxygen species; AIF, apoptosis-inducing factor; (Smac/DIABLO), Second mitochondria-derived activator of caspase/direct inhibitor of apoptosis-binding protein with low pI; AMPK, adenosine monophosphate-activated protein kinase.

Cytochrome c and Smac/DIABLO levels are also released when the mitochondrial membrane potential changes, and this results in the activation of the caspase cascade and the cleavage of poly [ADP-ribose] polymerase (PARP). 11 Interestingly, many of the bioactive compounds derived from four species of figs or Rauwolfia serpentina have inhibitory activity against PARP-1, indicating their potential as anticancer therapeutics. 12 13 Fisetin induced apoptosis in HCT-116 human colon cancer cells by upregulating proapoptotic proteins Bak and BIM and downregulating antiapoptotic proteins B cell lymphoma (BCL)-XL and -2. The activation of p53 promotes Bax's translocation to the mitochondria via a transcription-independent mechanism. By cleaving Caspase-8 and releasing cytochrome c and Smac/DIABLO through exogenous and endogenous processes, fisetin appears to induce apoptosis in HCT-116 cells ( Fig. 1 ). 14 15 16

### Activation of Autophagy Signaling Pathway by Fisetin

Autophagy is a self-degradative cycle by which broken cell parts are corrupted inside the cell and conveyed to the lysosome. 17 Induction, vesicle nucleation, elongation, retrieval, docking/fusion, and vesicle breakdown/degradation are the many steps of autophagy. Autophagy can be started by inhibiting mammalian target of rapamycin (mTOR) activity, which prevents Atg13's phosphorylation and causes a complex to form with Atg1 and Atg17. Phosphatidylinositol 3-kinase, or class III PI3K, is crucial for the initial stages of vesicle nucleation. Beclin-1 (Atg6), the UVRAG tumor suppressor gene, and myristylated kinase form a multiprotein complex that controls the protein's function (Vps15 or p150). Atg proteins such as the Atg12-Atg5 and LC3-II (Atg8-II) complexes control autophagosome development. Atg7 and Atg10 (E1- and E2-like enzymes, respectively) are required for the ubiquitin-like process that conjugates Atg12 to Atg5 during the vesicle elongation step.

**Fig. 2:**
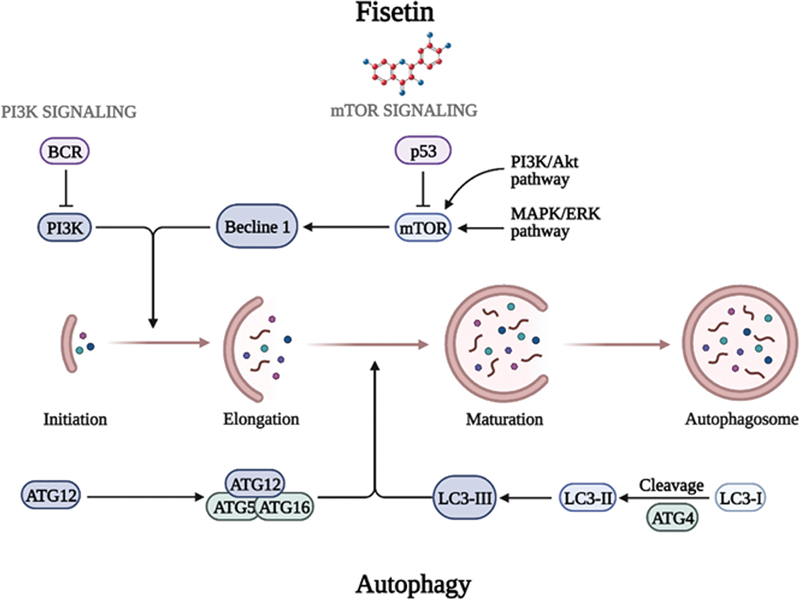
Activation of autophagy signaling pathways by fisetin. “↓” indicates “stimulate.” “⊥” indicates “inhibit”; solid line represents the action directly; dotted line represents the action through several processes. Abbreviations: The Beclin 1; protein is a central regulator of autophagy in mammalian cells; The ATG-ATG5:ATG7 complex is responsible for elongation of the phagophore in the autophagy pathway, With the help of ATG7 and ATG5, the ATG12-ATG5:ATG16L complex conjugates the C terminus of LC3-I to phosphatidylethanolamine in the phospholipid bilayer, allowing LC3 to associate with the membranes of the phagophore, becoming LC3-II. After formation of the autophagosome, the ATG12-ATG5:ATG16L complex dissociates from the autophagosome.

To create a substantial complex, the Atg12-Atg5 conjugate interacts noncovalently with Atg16. Cleaved LC3/Atg8 produces LC3-I, which is then conjugated to phosphatidylethanolamine (PE) by the E2-like enzymes Atg7 and Atg3 in a process resembling that of ubiquitin. A lipidated version of LC3 called LC3-II (autophagic vesicle-associated form) is created due to PE binding. An indication of autophagosome organization is LC3-II. Fig. 2 . Fisetin also blocks HepG2 cell autophagy via the PI3K/Akt/mTOR and AMPK pathways. 18

The cells are susceptible to fisetin, and the phagosomes were converted to double-layered membranes of autophagosomes through increasing expression levels of Atg proteins including Beclin-1and Atg-5,7, while LC3-I is converted to LC3-II and LC3-III. The inhibition of the Akt/mTOR/MAPK/extracellular signal-regulated kinases (ERK) signaling pathway assists in the accumulation of LC3-II, which suggests that the pathway is upstream of fisetin-induced autophagy. Once the autophagosome develops, its maturation is complete upon fusion with a lysosome to constitute an autophagolysosome.

## Cell Proliferation

Fisetin may influence signaling pathways relevant to cell survival, growth, and proliferation in both in vivo and in vitro. Fisetin can inhibit PCa cell growth and cause the death of PCa cells. 19 Fisetin could suppress lymph node carcinoma of the prostate (LNCaP), cwr22rv1, and PCa-3 cells, which are androgen-dependent and independent PCa cells but had no effect on normal prostate epithelial cells. 20 Fisetin has been shown to degrade the Wnt/β/β-catenin signal, and a decrease in melanocyte-inducing transcription factor (MITF) levels has adversely affected the development of human melanoma cells. 21 Fisetin targets the P70S6K and mTOR-protein kinases. 22 It suppresses the growth of human melanoma cells by physically attaching to these kinases, according to research on melanoma monolayer and a 3D model of melanoma skin equivalent. 23 Strong effects on several cancer cell types, including colon, cervical, and breast cancer cells, as well as their growth and spread.

## Cell Cycle Arrest

As our knowledge of the process of carcinogenesis has increased, research attention has shifted to the role of the cell cycle in the development of disease and malignant transformation. Therefore, it is believed that cell cycle-regulating molecules are viable therapeutic targets. Several drugs that target the cell cycle have also been evaluated in clinical trials with cancer patients. 24 This study found that fisetin triggered G1 phase arrest in LNCaP cells by activating WAF1/p21 and kip1/p27, followed by a reduction in cyclin D1, D2, and E as well as CDKs 2, 4, and 6. 20 In addition, screening study also examined the effects of flavonoids on the cell cycle of PCa cells. According to the results, fisetin triggered G2/M phase arrest in PC-3 cells while preventing G1 and G2/M phases in LNCaP cells. 25 In contrast, it triggered G1 phase arrest in 451lu melanoma cells, disrupted Wntβ/β-catenin signaling, and decreased survival rates in A431 human epidermoid carcinoma cells. 11

**Fig. 3:**
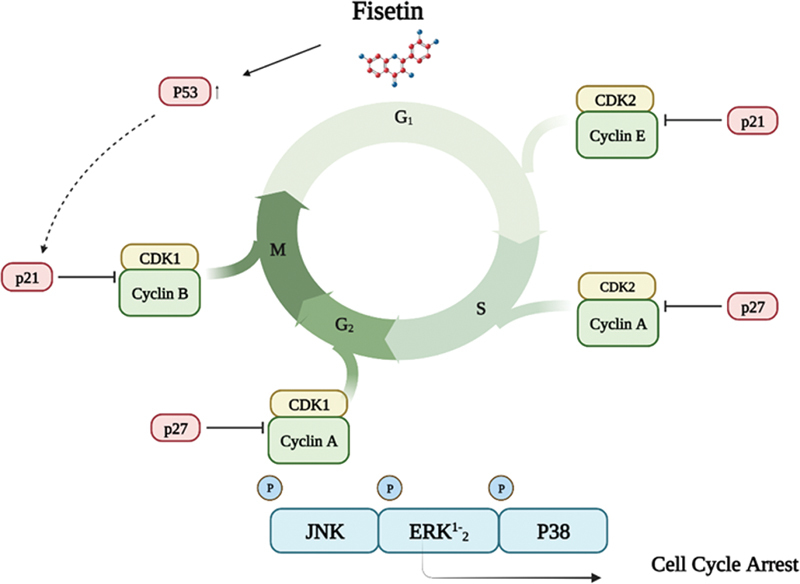
Regulation of the cell cycle regulatory proteins. (1) Modulation of cellular signaling pathways by fisetin-induced activation and arrests the cell cycle at various check points leading. Downregulation of p21, P27, and WAF1/KIP1, while upregulation of Cyclin E、Cyclin DA, resulting in arresting cells in M stage. (2) Inhibition of the expressions of CDK1、CKD、cyclin E、cyclin D1, causing to arrest cells at G2/M stage; activation of ALP to cells arrest at G2/M stage. Activation of ALP leads to cells arrest at G2/M stage. PC-3, A431,451lu melanoma cells which activated the G1,2/M as well as activation of Wnt/ β. “↓” indicates “stimulate.” “⊥” indicates “inhibit”; solid line represents the action directly; dotted line represents the action through several processes. Abbreviations: p21, protein of p21, a cyclin dependent kinase inhibitor; p27, protein of p53, a cyclin dependent kinase inhibitor; PCNA: proliferating cell nuclear antigen; CDK1, cyclin-dependent kinase1; CDK2, cyclin-dependent kinase2; ALP, alkaline phosphatase.

After treating HT-29 colon cancer cells with fisetin, the levels of WAF1 and p21 increased, the levels of cyclin E and D1 reduced, and the activity of CDK2 and CDK4 decreased. It shows that fisetin can block the cell cycle in HT-29 colon cancer cells and that this effect is partially explained by its impact on CDKs ( Fig. 3 ). 26

## Cell Migration and Invasion

According to a preliminary investigation, fisetin inhibits the expression of matrix metalloproteinases (MMP)-2 and -9 by blocking the PI3K/protein kinase B (AKT) and c-Jun N-terminal kinase signal transduction pathways reduces PC-3 cells' capacity for metastasis on the impact of PCa. 27 ERK signal is involved in fisetin-mediated suppression of the invasion and migration of the human lung cancer cell line A549. The fisetin decreased MMP-2 protein, messenger RNA (mRNA), and uPA levels through an ERK-dependent route. Furthermore, the NF of cells that received fisetin-B treatment and C-fos and c-jun nuclear levels drastically dropped. 28

**Fig. 4:**
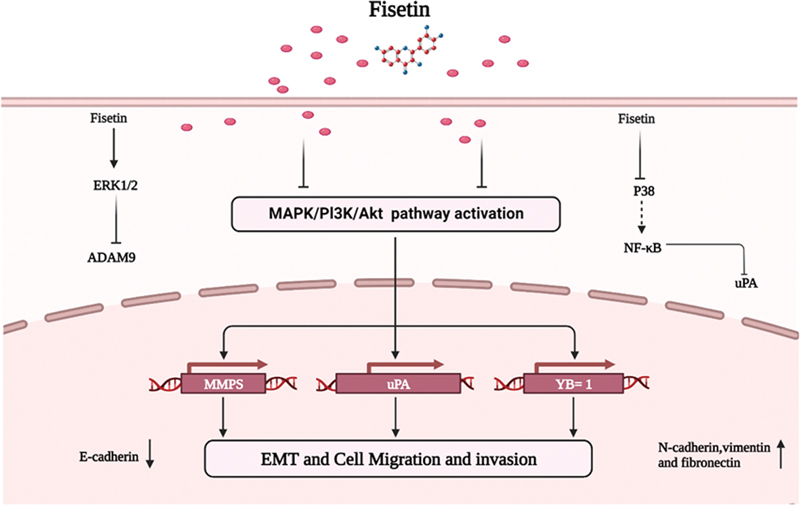
Effect of fisetin on some genes or enzyme. (1) Targeting at urokinase secretion and tubule formation of p38 cells, exhibiting the effect of anti-angiogensis and activation of NF-κB. (2) Inhibition of the expression of MAPK/P13K/AKT/JNK, promoter activity and uPA, MMPS and YB = 1 cell migration/invasion as well as EMT. Moreover, fisetin activated ERK1/2 that inhibited the ADAM9. “↓” indicates “stimulate” “⊥” indicates “inhibit”; solid line represents the action directly; dotted line represents the action through several processes. Abbreviations: p38, mitogen-activated protein kinases; NF-κB, Nuclear factor kappa light chain enhancer of activated B cells; uPA, a serine protease present in humans and other animals; The JNK, proteins belong to a class of proteins known as the MAPK, family of mitogen-activated protein kinase. They relay, amplify, and integrate signals from a diverse range of stimuli, both intra- and extracellular. ERK1/2: extracellular signal regulated protein kinase; AKT, protein Kinase-B.

Like this, treatment with fisetin in glioma gbm8401 cells can result in ongoing phosphorylation and activation of ERK1/2, blocking the metalloproteinase A disintegrin and a metalloprotease 9, which is linked to cell motility and invasion. 29 In addition, fisetin reduces the amount of uPA in human cervical cancer cells by blocking the nuclear factor-κB (NF-κB) signaling pathway dependent on p38 MAPK. Additionally, in cells treated with fisetin, tetradecylphorbol-13-acetyl reduce activated the p38 protein and reduced uPA production and secretion ( Fig. 4 ). 30

## Epithelial–Mesenchymal Transition

Epithelial–mesenchymal transition (EMT) is a developmental strategy that has been reserved throughout evolution. However, its activation can cause cancer cells to begin spreading metastatically. EMT involves transforming fully differentiated epithelial cells into poorly differentiated, migratory, and invasive mesenchymal cells.

This process is accompanied by a series of morphological alterations, the loss of intercellular contact, and matrix remodeling. 31 Latent membrane protein-1 (LMP1) of the Epstein–Barr virus can cause EMT and is connected to the metastasis of nasopharyngeal cancer cells. Fisetin can upregulate the epithelial marker E-cadherin, downregulate the mesenchymal marker vimentin, and drastically lower the EMT regulator twist protein level at noncytotoxic dosages, studies have revealed. This inhibits the migration and invasion of cne1-lmp1 cells. 32 While the nuclear translocation of YB-1 can be blocked by fisetin directly, EMT-induced expression of Y box-1 (YB-1) can accelerate the EMT in PCa. 33 As shown by kinetic studies and computational docking, Fisetin binds to the cold shock domain. The four chain residues interfere with the interaction between Akt and YB-1, preventing YB-1 from starting the EMT program. 33 To reduce the invasion of human melanoma cells, fisetin inhibits N-cadherin, vimentin, and fibronectin while boosting E-cadherin in vitro and xenograft tumors. 34

Research on the effects of fisetin on EMT is still in its early phases, and future studies will look closely at how fisetin impacts the EMT pathway ( Fig. 4 ).

## Role of Fisetin in Reactive Oxygen Species Reduction

Fisetin induces apoptosis in the human nonsmall lung cancer cell line NCI-H460, which causes DNA breakage, the growth of sub-G1 cells, depolarization of the mitochondrial membrane, and activation of caspases 9 and 3, which are involved in the production of intracellular reactive oxygen species (ROS). 35

Additionally, the down-regulation of birc8 and bcl2l2, as well as the accumulation of intracellular ROS, is associated with fisetin-induced apoptosis in human hepatoma Huh-7 cells. 36 This study proved that fisetin's induction of apoptosis in multiple myeloma cells needed the AMP-activated protein kinase (AMPK) pathway and ROS. 37 However, fisetin therapy has been linked to a reduction in ROS, according to other research. For instance, fisetin causes human promyelocytic leukemia cells to undergo apoptosis via activating Ca ^2+^ -dependent endonuclease, which is associated with reduced ROS levels. 38

## Synergism between Fisetin and Other Anticancer Drugs

Combination therapy is more efficient at inhibiting cell viability, preventing cell migration, and triggering apoptosis than fisetin alone. Melatonin inhibits COX-2/iNOS, NF-κB/P300, and cytochrome C-dependent apoptosis. In melanoma cells, a signaling pathway amplifies the action of fisetin. 39 Human melanoma cells that are BRAF mutants grow significantly more slowly when fisetin and the BRAF inhibitor sorafenib are applied together. Caspase-3 and PARP cleavage increased expression of Bax and Bak, and inhibition of Bcl-2 and Mcl-1 all contribute to an increase in apoptosis. 40 In a different study, the Hsp90 inhibitors geldanamycin and gibberellin increased the cytotoxicity of human colon cancer cells induced by fisetin by triggering the mitochondrial-dependent caspase-3 cascade and hastening the protein p53's phosphorylation. 41

Similarly, by controlling cell signal transmission, fisetin and hesperidin can cause apoptosis and cell cycle arrest in chronic myeloid leukemia cells. 42 Numerous signal pathways are regulated during fisetin-induced apoptosis. According to this study, fisetin activated the caspase-8 pathway through ERK1 and ERK2 to cause apoptosis in human cervical carcinoma HeLa cells. 15 43 Fisetin has been shown to block DR3-mediated NF-κB activation, induce apoptosis, and prevent invasion 44 in pancreatic cancer cell line AsPC-1. Effect of fisetin on NF-κB. The inhibition of B activation is mediated by regulating kinases, including rip, TAK1, and IKK κ B α degradation and nuclear translocation of p65. 45 Preacetone-induced apoptosis in human bladder cancer cells is upregulated by p53 and downregulated by NF-κB activity, changing the ratio of pro- to antiapoptotic proteins. Fisetin upregulated Bax and Bak expression while downregulating Bcl-2 and Bcl XL levels and activating the mitochondrial death pathway. 46 A comparable result was seen in rats treated with autologous bladder cancer. 47

Fisetin stimulates epidermal growth factor receptor/NF-κB -mediated apoptosis in cyclooxygenase COX-2 overexpressed human colon cancer cell line HT29. Blockage of the B signaling pathway. 48 Fisetin lowered the expression of COX-1 protein, downregulated COX-2, and decreased PGE2 production. Fisetin can also suppress the activity of Wnt/β signaling by inhibiting catenin and cytokine 4, which lowers the expression of target genes like cyclin D1 and MMP-7. 48 In addition, another method that, fisetin (HSF1), promotes apoptosis by regulating the transcription factor heat shock factor 1, which controls the expression of heat shock proteins (HSPs). 49 Fisetin is a strong HSF1 inhibitor that blocks HSF1 from binding to the hsp70 gene promoter. Fisetin also inhibited the antiapoptotic proteins Bcl-2, BCL XL, and Mcl-1's chaperones HSP70 and bag3, demonstrating the existence of a new mechanism for regulating apoptosis. 49

## Research Progress of Fistein and Its Antitumor Effects on Animal Experiments

Animal experiments play a critical role in preclinical research as they provide a platform for investigating the efficacy and safety of potential therapeutic agents before moving to human trials. We have extensively reviewed the existing literature on the in vivo effects of fisetin in animal models of cancer. These studies have significantly contributed to our understanding of fisetin's potential as a new therapy for different types of cancer.

For instance, Kim demonstrated that fisetin induced apoptotic cell death and endoplasmic reticulum (ER) stress in human endometrial cancer cells. 50 Ragelle et al developed a nanoemulsion formulation of fisetin that improved its bioavailability and antitumor activity in mice. 51 Li et al conducted both in vitro and in vivo experiments and found that fisetin remarkably inhibited colorectal cancer cell proliferation and migration, induced cell cycle arrest and apoptosis, and suppressed tumor growth in tumor-bearing mice. 52

Additionally, Jia et al investigated the in vivo antitumor effect of fisetin in a pancreatic cancer model and identified the enhancement of the AMPK/mTOR signaling pathway after fisetin treatment. 53 Li et al explored the effects of fisetin in a metastatic breast cancer xenograft model and observed significant inhibition of primary tumor growth and reduction in lung metastasis. 54

These animal studies, along with several others in the literature, collectively provide a significant amount of information regarding the in vivo effects of fisetin in animal models of cancer. They enhance our understanding of fisetin's mechanisms of action, highlight its antitumor activity, and support its potential as a new therapy for different types of cancer.

We acknowledge that while animal studies provide valuable preclinical evidence, there are limitations and challenges associated with extrapolating these findings to humans. Factors such as species differences, dosing regimens, treatment duration, and potential adverse effects need to be carefully considered and discussed when interpreting the results. We addressed these limitations in the paper, providing a balanced perspective on the potential of fisetin as a therapeutic agent for cancer treatment.

These studies, along with others in the literature, provide valuable insights into the in vivo effects of fisetin and its potential as an antitumor therapy. By referring to relevant scientific literature and conducting a thorough literature search, we have identified specific studies that delve deeper into the in vivo effects of fisetin in various tumor models, enhancing our understanding of its therapeutic potential.

Considering the limitations and challenges associated with in vivo studies is important as well. Factors such as species differences, dosing regimens, treatment duration, and potential side effects need to be carefully considered and discussed when interpreting the findings. By acknowledging these limitations, we can provide a more comprehensive and balanced perspective on the potential of fisetin as a therapy.

In conclusion, this information will strengthen our argument, provide mechanistic insights, and offer valuable information on the translational potential of fisetin for human clinical trials. We provided the appropriate references for the studies mentioned to ensure the scientific validity and credibility of our findings. 50 51 52 53 54

## Fisetin and Cancer

Chemoprevention is a new, alluring, and creative approach to cancer treatment. While fruits and vegetables are a great source of numerous cofactors, vitamins, and minerals, flavonoids, a phytochemical can uniquely target several important cellular processes implicated in cancer growth. The most prevalent polyphenols found in human food are called flavonoids, which are further divided into flavonols, flavones, isoflavones, anthocyanidins, theaflavins, and thearubigins. Numerous studies have shown flavonoids to have antioxidant, anti-inflammatory and chemopreventive characteristics in different cancers. 10 16 55

**Fig. 5:**
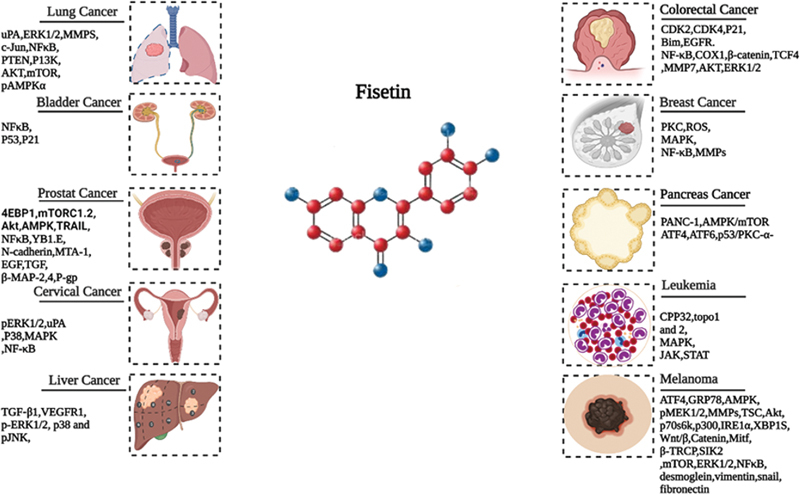
Fisetin's molecular targets in different cancers.

The naturally occurring flavone 3,3′, 4', and 7-tetrahydroxyflavone is also known as fisetin. Strawberries, apples, persimmons, grapes, onions, and cucumbers are common foods containing fisetin. 56 Fig. 5 illustrates how various molecular and signaling pathways are impacted by fisetin depending on the kind of cancer. Among its many biological functions, fisetin has recently been studied for its potential to fight cancer, making it a viable drug for cancer treatment and prevention. This paper describes its cellular actions to clarify fisetin's preventive and therapeutic potential against various malignancies ( Fig. 5 ).

Fisetin binds to and engages with several molecular targets to connect with various cellular targets. Numerous cellular processes are regulated by fisetin. Fisetin interferes with Wnt signaling, which stops the cell cycle. Inhibiting the YB-1 binding protein limits the invasion and migration of tumors by suppressing the EMT cells. Fisetin reduces cell survival signaling by physically engaging with the mTOR protein, which accounts for the inhibitory effects of the compound on the growth and division of cells. Fisetin attaches to microtubules, disturbs their dynamics, and stabilizing effects much better than paclitaxel. However, mTOR and NF-κB appear to be the most frequently impacted pathways in all malignancies.

## Fisetin and Lung Cancer

The most common cancer-related cause of death in the world is lung cancer. It causes 27% of all cancer-related deaths and 13% of all diagnosed cancers. 1 The emergence of cisplatin resistance, a commonly used chemotherapeutic medication, is a significant barrier to the chemotherapy of nonsmall cell (NSC) lung cancer. In lung cancer cells, fisetin has demonstrated antiproliferative, apoptosis, and antiangiogenic activities. In A549-CR lung cancer cells, fisetin has been shown to overcome the developed isolation resistance. Compared with cells treated with fisetin and cisplatin separately, cells treated with the combination of the two drugs showed a significant loss of cell viability and induction of apoptosis, potentially through the inactivation of MAPK pathways and the inhibition of survivin expression. 57

Fisetin has been demonstrated to activate mitochondrial-mediated mechanisms to cause apoptosis in NSC lung carcinoma. By decreasing the expression of Bcl-2 and increasing the expression of Bax, fisetin caused DNA fragmentation, ROS production, and death in NCI-H460 cells. The cleavage of caspase-9 and caspase-3 caused by fisetin therapy boosted caspase-3 activation. 35 55 Similar to how benzo(a) pyrene (B(a)P)-induced lung cancer in vivo, fisetin supplementation reduced mitochondrial dysfunction and produced apoptosis by upregulating the Bax/Bcl-2 ratio, causing the cytochrome-c release and activating caspase-9, and caspase-3 leading to apoptotic cell death. 58 Fisetin, 25 mg per kg of body weight] B(a)P treatment for Swiss Albino mice reduced histological lesions, levels of lipid peroxidation, and altered enzymatic and nonenzymatic antioxidants. 59 We are sure that fisetin therapy (5–20 µM) prevents A549 NSC lung cancer cells from growing and forming colonies. Fisetin phosphorylated AMPK, which in turn activated the tumor suppressor PTEN and adversely regulated protein synthesis. Additionally, fisetin blocked the PI3K/Akt/mTOR signaling pathway in NSC lung cancer cells. Fisetin is a promising prospective medication for therapeutic intervention in lung cancer, according to studies including this system's inhibitors that have entered preclinical and clinical stages. 60

Using a Matrigel plug experiment, it was demonstrated that fisetin in conjunction with CPA inhibited angiogenesis. In contrast to animals treated with fisetin or CPA alone, the combination of fisetin (223 mg/kg) and CPA (30 mg/kg) exhibited significant suppression of tumor growth (92%) in Lewis lung carcinoma (LLC)-bearing mice. The first proof that fisetin had antiangiogenic and anticancer properties in mice carrying LLC was presented by this study. 61 Another study found that fisetin inhibits A549 lung cancer cells' adhesion, migration, and invasion via downregulating uPA, ERK1/2, and MMP-2. Moreover, NF-κB, c-Fos, c-Jun, and AP-1 nuclear levels were also lowered by fisetin treatment, and NF-κB binding was inhibited. Together, fisetin showed positive in vivo and in vitro outcomes against lung cancer.

## Fisetin and Prostate Cancer

The most common male malignancy is PCa, with an estimated 220,800 new cases yearly. 1 Treatment and prevention of PCa with fisetin are active areas of research. Studies in cell culture demonstrate that fisetin inhibits the proliferation of human PCa cells. Previous studies have demonstrated that fisetin administration of LNCaP cells inhibited PCa by causing G1-phase cell cycle arrest, altering the CKI- cyclin-CDK network, and inducing apoptosis. 20 In LNCap cells, fisetin promoted apoptosis and cell cycle arrest; in athymic nude mice implanted with androgen receptor-positive 22R one cell, fisetin reduced androgen signaling, and tumor formation. 62 By hypophosphorylated 4E-binding protein-1, fisetin decreased cell proliferation. It also caused autophagic cell death in PCa cells by inhibiting mTORC1 and mTORC2. Fisetin functions as a dual mTORC1/C2 inhibitor, activating the mTOR repressor TSC2.

These effects are often linked to the suppression of Akt and activation of AMPK. 63 TRAIL is critical for the body's defense against malignant cells. Fisetin made the androgen-independent DU145 and PC3 PCa androgen-independent LNCaP cells susceptible to TRAIL-induced apoptosis. Furthermore, compared with androgen-dependent LNCaP cells, the cytotoxic and apoptotic effects of TRAIL combined with fisetin are the least in androgen-independent PCa cell lines. Fisetin's ability to prevent NF-κB activation in LNCaP cells enhanced TRAIL's ability to cause apoptosis. These results support the hypothesis that NF-κB downregulation sensitizes PCa cells to TRAIL in vitro. 64

Fisetin has been proven to block YB-1, a crucial transcription factor encouraging EMT in PCa. In contrast to E-cadherin, a hallmark for EMT, YB-1 is overexpressed in PCa. Both in vitro and in vivo, forced YB-1 expression caused a mesenchymal phenotype during PCa development. Fisetin has been described as an inhibitor of YB-1 phosphorylation and MTA-1 production because it interacts with the cold shock domain of the YB-1 protein. Fisetin also prevents EMT and YB-1 phosphorylation triggered by EGF and TGF- in PCa cells. 33 Both in vitro and in vivo tests showed that TMFol, a structural homolog of fisetin and quercetin, had more chemopreventive activity than fisetin alone. The proliferation of 22Rv1, TRAMP C2, PC-3, and LNCaP cells decreased by TMFol. Additionally, it inhibited the growth of tumors in nude mice containing TRAMP C2 and 22Rv1 cells. 65 As recently demonstrated, Fisetin alters microtubule dynamics in PCa cells by binding to β-tubulin and promoting tubulin polymerization. Fisetin reduces the proliferation, invasion, migration, viability, colony formation, and P-gp protein in multidrug-resistant NCI/ADR-RES cells while arresting cells in the G2/M phase. These in-vitro findings confirm fisetin's role as a microtubule-targeting drug. They indicate that it has the potential to be developed as an adjuvant to medicines that also target microtubules. 66

Collectively, these studies offer enough proof that fisetin could be turned into a powerful PCa treatment by focusing on various routes.

## Fisetin and Melanoma/Skin Cancer

Melanoma is the most lethal type of skin cancer since it has a high chance of metastasizing. 1 Fisetin's ability to prevent human melanoma by interfering with Wnt/β /β-catenin/MITF signaling has been described for the first time. In-vitro studies revealed potential mechanisms for fisetin-mediated regulation of Wnt/β signaling in 451Lu human melanoma cells, including a decline in β-catenin levels, an increase in β-TrCP, and a drop in MITF mRNA and protein levels. Fascinatingly, the in-vitro results were replicated in in-vivo research, demonstrating that fisetin greatly reduced tumor growth in 451Lu melanoma xenografts, which was connected to lower MITF levels. 21 55 Furthermore, a different study showed that the mono-methyl analog of fisetin, 4 MF, is a potent SIK2 inhibitor and significantly increases melanogenesis in B16F10 melanoma cells.

CREB-mediated transcription of TORC1 by modifying SIK2 signaling with 4 MF. 67 According to research on nonmelanoma skin cancer, fisetin increased the expression of Bax, Bak, and Bad in A431 cells, which hindered proliferation and caused apoptosis. Fisetin administration also caused G2/M arrest, Bcl-2, Bcl-xL, and Mcl-1 protein modification, disruption of mitochondrial potential, activation of caspases, and cleavage of PARP. 11 In the SKH-1 mouse skin cancer model, a different group looked at the photochemopreventive impact of fisetin for treating UVB-induced skin malignancy.

According to their research, topical application of fisetin (250 and 500 nmol) to the skin of SKH-1 hairless mice after exposure to UVB light causes a significant reduction in leukocyte infiltration, inflammatory markers (MPO, COX2, and PGE2), cytokines (TNF-, IL-1, and IL-6), and proliferation markers (PCNA and cyclin D1). Additionally, PI3K/AKT/ NF-κB signaling, which is linked to UVB-induced inflammation, cell survival, and proliferation, was suppressed by fisetin. After UVB exposure, fisetin was found to have no negative effects on mouse skin and to boost the expression of the p53 and p21 proteins. 68

A significant regulator of melanoma cell invasion and a possible target for melanoma treatment and prevention is the MAPK (BRAF-MEK-ERK) pathway. Inhibition of cell invasion was seen in melanoma cells A375, SK-MEL-28, RPMI-7951, SKMEL-119, and Hs294T after treatment with fisetin (5–20 µM). It was discovered that BRAF-mutated cells were more sensitive because MEK1/2 and ERK1/2 were not phosphorylated as much. The NF-κB signaling pathway was less activated due to fisetin's inhibition of IKK activation. As evidenced by a decrease in mesenchymal markers (N-cadherin, vimentin, snail, and fibronectin) and an increase in epithelial indicators, fisetin also accelerated the phenotypic shift of melanomas from mesenchymal to epithelial (E-cadherin and desmoglein). Fisetin also suppresses the growth of A375 human melanoma cells in monolayer and three-dimensional preparations. With the downregulation of mTOR and Akt and the overexpression of TSC, fisetin reduced melanoma progression in a 3D melanoma skin model. Fisetin suppressed the growth of A375 and 451Lu melanoma cells by more strongly binding to p70s6K than mTOR. 69 Fisetin's improved anticancer efficacy was shown in the melatonin and fisetin combination therapy research. Compared with fisetin alone, the combination dramatically reduced viability, motility, and colony formation and generated higher levels of apoptosis in melanoma cells. These effects were connected to the stimulation of cytochrome-c/caspase-dependent apoptosis and the suppression of COX-2 and iNOS production mediated by p300/ NF-κB. 39 Fisetin has been shown to play a role in human melanoma ER stress and the activation of intrinsic and extrinsic apoptotic pathways. In A375 melanoma cells, fisetin increased NO production while inhibiting ROS. Apoptosis caused by fisetin (20–80 µM) was accompanied by brief autophagy and the production of ER stress, which was shown by elevated levels of IRE1 α, XBP1s, ATF4, and GRP78 in A375 and 451Lu cells. Failure of AMPK silencing to stop cell death shows that both AMPK-dependent and -independent pathways are involved in fisetin-induced cytotoxicity. 70

In mice implanted with BRAF-mutant melanoma cells, a different combinatorial method utilizing fisetin and sorafenib (an RAF inhibitor) has recently shown suppression of melanoma cell proliferation, induction of apoptosis, and prevention of tumor growth. The combo treatment increased apoptosis and caused caspase-3 and PARP to be cleaved, phosphorylated, Bax and Bak to be expressed, Bcl-2 and Mcl-1 to be inhibited, PI3K to be expressed less frequently, and MEK1/2, ERK1/2, AKT, and mTOR to be phosphorylated. Compared with fisetin and sorafenib alone, fisetin treatment significantly reduced tumor growth and inhibited the MAPK/PI3K pathways in A375 and SK-MEL-28 cell xenografts, indicating that combination therapy is superior to monotherapy. 71 Furthermore, by lowering MMP-2 and MMP-9 proteins in melanoma cell xenografts, it has been demonstrated that combining fisetin with sorafenib successfully reduced EMT and enhanced the antimetastatic efficacy of sorafenib. It showed fisetin's ability to treat melanoma independently and with other proven treatments.

## Fisetin and Colorectal Cancer

There are notable international variations in the distribution of colorectal cancer, the third most prevalent cancer in women and the fourth most common cancer in males. 72 In HT-29 human colon cancer cells, fisetin (0–60 µM) was identified as dose-dependently blocking CDK activity, resulting in cell cycle arrest. Cell cycle progression began to cut back by fisetin at 8 hours, followed by an arrest in the G2/M phase at 72 hours. Fisetin therapy increased p21 (CIP1/WAF1) levels and lowered cyclin-E and cyclin D1 levels, increasing CDK2 and CDK4 activities. Additionally, altering CDK activity can account for the suppression of cell cycle progression in HT-29 cells after fisetin therapy. 26 55

A subsequent investigation characterized the exact mechanism of fisetin-mediated apoptosis in HCT-116 colon cancer cells. In HCT-116 cells, fisetin (5–20 µM) caused DNA condensation, PARP breakage, and caspase-9, 7, and 3 activations. Moreover, pro-apoptotic proteins Bak and Bim were increased, whereas antiapoptotic proteins Bcl-xL and Bcl-2 were downregulated, which caused Bax to translocate into the mitochondria. Additionally, fisetin raised p53 protein levels, while small interfering RNA-mediated reduction of p53 expression decreased the effects of fisetin on Bax translocation to the mitochondria, releasing mono- and oligonucleosomes in the cytoplasm and PARP breakage. Fisetin's ability to stimulate apoptosis by activating caspases and inducing p53, which causes the translocation of Bax to mitochondria, was necessary to demonstrate in this work. 14

It is known that Wnt/β signaling and COX-2 overexpression contributes to colorectal cancer. Fisetin (30–120 µM) inhibits COX-2 and the Wnt/β/ EGFR/ NF-κB signaling pathways, which cause apoptosis in colon cancer cells. In HT-29 and HCT116 colon cancer cells, fisetin caused apoptosis, downregulated COX-2 protein expression without altering COX-1, and decreased prostaglandin E2 release. In addition, fisetin therapy reduced levels of β-catenin, TCF-4, cyclin D1, and MMP-7, which suggests a potential function for the drug in colon cancer prevention. 48 A different investigation discovered that fisetin treatment could radiosensitize human colorectal cancer cells that are resistant to radiotherapy. Fisetin pretreatment of p53-mutant HT-29 cells increased their radiosensitivity by creating an accumulation of cells in the radiosensitive G2/M phase, reducing their ability to repair DNA and thereby raising the number of double-strand breaks brought on by radiation. Additionally, radiation-induced proapoptotic p38 MAPK activation and eventual suppression of prosurvival signals were enhanced by fisetin pretreatment. 73

Compared with fisetin treatment alone, a recent combinatorial study found that 10 to 20 M NAC increases fisetin-mediated apoptosis in COLO25 colon cancer cells. In COLO25 cells, the combination of fisetin and NAC treatment elevated cleaved caspase-3, and PARP lowered mitochondrial membrane potential and induced caspase-9. Other cells, including HCT-116, HT-29, and HCT-15, showed NAC sensitization to fisetin-induced apoptosis, suggesting a unique method to treat colon cancer. 41 Fisetin is difficult to administer intravenously since it is poorly soluble in water. By creating nanoassemblies of polymeric micelles that can encapsulate fisetin, a unique technique was used to solve this problem, resulting in an increased therapeutic impact on colon cancer. A sustained and protracted in-vitro release, increased cytotoxicity, cellular uptake, and apoptosis were all seen with the polymeric micelle encapsulation. Additionally, fisetin micelles demonstrated higher tumor apoptosis, proliferation inhibition, and antiangiogenesis effects. 74

## Fisetin and Bladder Cancer

If not properly treated, bladder cancer is a complex condition with significant rates of morbidity and fatality. The key to a successful outcome is early identification with individualized therapy and follow-up, with awareness of hematuria as the primary presenting symptom of the greatest importance. 75 76 Fisetin altered the ratio of pro- and antiapoptotic proteins in human bladder cancer by upregulating p53 and downregulating NF-κB activation. According to the study's findings, fisetin prevents the growth of T24 and EJ cells by triggering apoptosis and impeding the advancement of the cell cycle in the G0/G1 phase by considerably raising the levels of the proteins p53 and p21 and lowering those of cyclinD1, cyclinA, CDK4, and CDK2. This in-vitro investigation revealed that p53 activation and NF-κB suppression are crucial for bladder cancer cells to undergo fisetin-induced apoptosis. 46

According to a subsequent in-vivo investigation employing a rat bladder cancer model produced by MNU, p53 activation and NF-κB suppression are key factors in the bladder cancer apoptosis caused by fisetin. Furthermore, fisetin prevented tumor development and bladder carcinogenesis in rats given MNU without causing harm. 77 These results showed that fisetin is an effective chemopreventive agent in vivo and imply that fisetin may be developed as a thriving chemopreventive agent for bladder cancer.

## Fisetin and Breast Cancer

The most common cancer in women is breast cancer. Although surgery can cure most benign breast cancers, one-quarter have a latent and sneaky nature that spreads quickly despite growing slowly. 78 Fisetin's cytotoxicity and apoptotic effects on MCF-7 and MDA-MB-231 breast cancer cells were thoroughly examined. In MCF-7 cells lacking caspase-3, fisetin was found to have anticancer properties. However, fisetin also caused a novel type of atypical apoptosis in MCF-7 cells and prompted plasma membrane rupture, mitochondrial depolarization, caspase-7, 8, and 9 activations, and PARP cleavage. The absence of caspase-3 in MCF-7 cells was the cause of these unusual characteristics of apoptosis. Fisetin also decreased autophagy, accelerating cell death in MCF-7 cells. 79 In breast cancer cells (4T1 and JC cells), fisetin increased HO-1 mRNA and protein expressions, elevated Nrf2 expression, and abolished the HO-1 expression, whereas HO-1 expression was mediated by upregulation of the transcription factor Nrf2. In addition, fisetin reduced MMP-2 and MMP-9 enzyme activity and gene expression for both mRNA levels and protein. 16 These few pilot studies imply that fisetin's bioactive properties could effectively fight against breast cancer.

## Fisetin and Leukemia

Only a few studies have examined the efficacy of fisetin in treating leukemia. Leukemia is a blood and bone marrow cancer that can be classified into four main types based on the kind of cell and rate of growth: acute lymphocytic (ALL), chronic lymphocytic (CLL), acute myeloid (AML), and chronic myeloid (CML). With a projected 54,270 new cases and 24,450 fatalities, leukemia remains a health problem. 1

An investigation was conducted on the biological effects of seven structurally related flavonoids on the human leukemia cell line HL-60. Fisetin was the most cytotoxic flavonoid, while wogonin and fisetin worked best as an apoptosis inducer. The interaction resulted from rapid and transient induction of caspase-3/CPP32, degradation of PARP, and reduction of Mcl-1, an antiapoptotic protein. The combo therapy did not affect Bcl-2, Bcl-xL. Combination therapy reduced ROS production in apoptosis while increasing Ca (2 + )-dependent endonuclease activity. A fascinating connection between flavonoids encourage apoptosis and the antioxidant capabilities of fisetin and wogonin. 38 The number of frequently used cancer chemotherapy drugs particularly target DNA topoisomerases (topo). These drugs induce topo-DNA complexes with either topo I or topo II, ultimately resulting in cell death. 80 It has been discovered that patients taking some topo II inhibitors have a higher risk of developing leukemia. The effects of many flavonoids, including fisetin, were investigated in K562 human leukemia cells at various exposure levels and periods. Both enzymes were catalytically inhibited by fisetin but could not cause topo I- or topo II-DNA complexes to form. These results show that fisetin blocks DNA topoisomerases I and II in leukemia cells. 80

Fisetin and hesperetin's function in human HL-60 acute promyelocytic leukemia cells and their mechanism(s) of action have recently come to light. Hesperetin and fisetin induced G2/M arrest, increased caspase-3 activity, inhibited cell growth, changed mitochondrial potential, and aided in promoting death. In microarray gene profiling of the treated cells, some important biological pathways, including MAPK, DNA binding signaling pathways, and genes involved in cell proliferation, division, and death, were discovered. Another analogous study demonstrated the combined effects of fisetin and hesperetin on human K562 CML cells. The combination treatment drastically altered genes involved in replication, transcription, translation, apoptosis, cell division, cell proliferation, and many other cellular processes. Microarray gene profiling studies identified the JAK/STAT pathway, KIT receptor, and growth hormone receptor signaling genes as potential candidates for the fisetin-hesperetin combo for targeted CML therapy. 81

## Fisetin and Cervical Cancer

Cervical cancer affects almost 500,000 women worldwide each year, making it a serious health problem. 82 An initial experiment found that fisetin-activated caspase-3 and -8 and cleaved PARP cause human cervical cancer HeLa cells to undergo apoptosis. Fisetin administration also caused pERK1/2 to become permanently activated. Using ERK1/2 inhibitors or transfecting cells with mutant ERK1/2 expression vectors reversed fisetin-induced apoptosis. This study showed that fisetin activates ERK1/3, a component of the caspase-3/caspase-8 pathway, to cause apoptosis in HeLa cells. In tumor xenograft testing on mice, fisetin significantly slowed tumor growth by reducing the growth of tumors. 30

According to a different study, Fisetin inhibits the migration and invasion of the human cervical cancer cells SiHa and CaSki, which provide significant support for the hypothesis that fisetin inhibits aggressive behaviors by downregulating the expression of the uPA gene by inhibiting the p38 MAPK-dependent NF-κB signaling pathway. 43 A novel combination technique using fisetin and sorafenib on human cervical cancer cell lines demonstrated a potent anticancer impact both in-vitro and in-vivo. The combined therapy led to apoptosis in HeLa and SiDR5-treated HeLa cells through the activation of caspase-3 and -8, followed by a significant loss of mitochondrial potential. Additionally, studies in animals using a HeLa xenograft model demonstrated that sorafenib treatment administered alone was unquestionably inferior to that administered in conjunction with fisetin. Future cervical cancer clinical trials may consider using this fisetin and sorafenib therapy combination. 40

## Fisetin Therapeutic Mechanism Potential

Cancer is a complex disease that requires several signaling pathways to survive and is one of the leading causes of death. Single-agent targeted therapy has very seldom been able to treat cancer in patients. A drug that can effectively target a variety of aberrant proteins and pathways can prevent the growth and spread of cancers. In preclinical studies, the dietary flavonoid fisetin has shown an extraordinary ability to target several unregulated proteins and signaling pathways and regulate various cell functions against various cancers. Fisetin prevents Wnt/β signaling from functioning, which halts the cell cycle. By preventing the EMT, inhibiting the YB-1 binding protein restricts cancer cell invasion and migration. Fisetin inhibits cellular growth and proliferation by physically interacting with the mTOR protein, which reduces signals vital to cell survival. When fisetin binds to microtubule dynamics, it modifies them and acts as a stabilizing agent with significantly better outcomes than paclitaxel. Before starting valid clinical studies, research the toxicological profile of fisetin in great detail. Unfortunately, there are currently no fisetin toxicity data to support its prospective usage in humans. Before phase I and II trials are started, it is also necessary to thoroughly research the pharmacokinetic and bioavailability difficulties.

Many people are interested in the discussion around cancer chemoprevention since dietary components show promise in preclinical studies but have no impact in clinical trials. Several criticisms downplay the significance of phytochemicals based on their numerous biological impacts, targets, and signaling pathways. However, we firmly believe that cancer chemoprevention has many potentials if it is properly modeled to give an efficient alternative strategy for managing cancer, especially for high-risk persons. 83 One of the most significant obstacles to developing targeted medicines is the emergence of drug resistance. The solution to the issue of resistance may lie in combining targeted therapy with more conventional ones. However, the development of FDA-approved anticancer medications continues to be hampered by problems with toxicity and high treatment costs. Fisetin exhibits significant promise as an anticancer treatment, and there is enough convincing preclinical data to support clinical trials using it alone or in conjunction with other anticancer medications.

## Challenges and Future Directions in Studying Fisetin for Its Antitumor Effects

Studying natural compounds like fisetin for their therapeutic potential presents both opportunities and challenges. Natural products often exhibit complex chemical profiles, making it crucial to identify and characterize the specific active components and their mechanisms of action. 84 Additionally, optimizing the formulation and delivery of natural compounds can be challenging due to their low bioavailability and rapid metabolism. 85 86 Therefore, future research should focus on enhancing the bioavailability and stability of fisetin, potentially through novel delivery systems or formulation approaches. 86

Furthermore, understanding the pharmacokinetics and toxicity profiles of fisetin is essential for its clinical translation. Preclinical studies have indicated the potential efficacy of fisetin in inhibiting tumor growth and inducing apoptosis. 87 88 However, further investigations are needed to elucidate the long-term effects, optimal dosing regimens, and potential adverse effects associated with fisetin treatment. 88

In addition to addressing the challenges, exploring future research directions is crucial for advancing the field. Investigating the synergistic effects of fisetin with other therapeutic agents or treatment modalities could enhance its antitumor efficacy. 84 Moreover, investigating the potential role of fisetin in combination with immunotherapy or targeted therapies may offer promising treatment strategies. 89

To support our discussion, we have included relevant scientific references, providing a systematic review on the challenges and opportunities in developing fisetin as an anticancer agent and offering valuable insights into the current state of the field. 84 McMichael 90 discuss the challenges and opportunities in natural products research, emphasizing the importance of rigorous scientific investigation for validation. 91 Furthermore, van Wyk and Albrecht highlight the considerations in studying natural products and the need for preclinical and clinical studies. 87 Fernández-Arroyo et al discuss the bioavailability challenges of polyphenols, which are relevant to fisetin. 86 Finally, Singh et al provide insights into the potential mechanisms of action and suggest future research directions for fisetin. 88

One challenge is related to the bioavailability of fisetin, as it is known for its poor solubility and limited systemic absorption. This can affect its therapeutic efficacy and necessitates the exploration of novel drug delivery systems or formulation strategies to improve its bioavailability and ensure optimal therapeutic concentrations are achieved at the target site

Another challenge lies in the complex and multifaceted nature of the molecular mechanisms underlying fisetin's antitumor effects. Fisetin has been reported to modulate multiple signaling pathways involved in cell proliferation, apoptosis, angiogenesis, and inflammation. Elucidating the precise molecular targets and pathways affected by fisetin will require further investigations using advanced techniques such as proteomics, genomics, and metabolomics.

Furthermore, the heterogeneity of cancer types and individual patient responses presents a challenge in studying fisetin's antitumor effects. Different cancer types may exhibit variable sensitivity to fisetin, and individual patient characteristics, including genetic and molecular profiles, may influence treatment outcomes. Therefore, future studies should aim to investigate the effects of fisetin in a wide range of cancer models representing different cancer types to assess its efficacy and potential limitations in specific contexts.

Regarding future directions for research, several areas warrant further investigation. One important direction is the exploration of combination therapies involving fisetin. Combining fisetin with other anticancer agents or conventional therapies may lead to synergistic effects and improved treatment outcomes. Further exploration of combination strategies can help optimize treatment regimens and overcome potential resistance mechanisms.

Another direction is the investigation of fisetin in specific cancer types and subtypes. Fisetin's effectiveness may vary among different cancer types due to their distinct molecular characteristics. Therefore, it is important to conduct studies in a variety of cancer models representing different subtypes to identify those where fisetin demonstrates the most promising antitumor effects.

Additionally, understanding the impact of fisetin on the tumor microenvironment and its immunomodulatory properties is an emerging area of research. Fisetin has been reported to modulate immune responses and affect the tumor microenvironment, potentially influencing tumor growth and metastasis. Exploring fisetin's effects on immune cells, inflammatory mediators, and tumor-immune interactions can provide insights into its broader therapeutic potential and guide the development of immunotherapeutic strategies.

## Conclusion and Perspective

Numerous studies have demonstrated that fisetin may inhibit or delay the growth of many cancer cells. In addition, fisetin may induce apoptosis via both mechanisms. Upon application of fisetin, phagosomes were changed into autophagosomes with double-layered membranes due to the inhibition of the Akt/mTOR signaling pathway; this indicates that the route is upstream of fisetin-induced autophagy. Fisetin inhibits several cancer-related pathways and prevents cancer by promoting apoptosis. As an adjunct to other chemotherapy drugs, fisetin can be used to treat cancer. As our understanding of fisetin's mechanism of action increases, these therapy strategies will boost its efficacy in treating cancer. Numerous studies have shown that adding fisetin to chemotherapy can increase effectiveness. Furthermore, combinations may reduce the effective dose of therapeutic drugs, thereby reducing dose-related side effects. However, before it can be used safely with other drugs, fisetin's influence on these pharmaceuticals' pharmacological metabolism must be studied further. The potential chemical synergy and medication combination's onset mode and its impact on the molecular route are not fully understood, and future studies to clarify this are required.
